# Spleen Involvement at Diagnosis of Multiple Myeloma: A Case Report and Literature Review

**DOI:** 10.1002/cnr2.70160

**Published:** 2025-03-12

**Authors:** Alessia Fiorini, Maria Gabriela Chavez, Valentina Panichi, Marco Dell'Aquila, Valentina Ranucci, Daniele Remotti, Michela Tarnani, Marco Montanaro, Roberto Latagliata, Alessandro Andriani

**Affiliations:** ^1^ UOC Haematology ASL Viterbo—Santa Rosa Hospital Viterbo Italy; ^2^ Service of Transfusional Medicine ASL Viterbo—Santa Rosa Hospital Viterbo Italy; ^3^ UOC Clinical Diagnostic, Cytofluorimetric Laboratory ASL Viterbo—Santa Rosa Hospital Viterbo Italy; ^4^ UOC Anatomopathology ASL Viterbo—Santa Rosa Hospital Viterbo Italy

## Abstract

**Background:**

Multiple myeloma (MM) is more often characterized by clonal plasma cell proliferation restricted to the bone marrow. However, a small percentage of patients with MM develop extramedullary disease (EMD): this type of localization is found in 1.7%–4.5% of the newly diagnosed MM (ND/MM) and in 3.4%–10% of patients with relapsed or refractory disease (RR/MM) and seems to have a bad prognostic impact. In the present report, we describe a very rare case of splenic involvement in a patient with ND/MM.

**Case:**

A 72‐year‐old female was referred to Santa Rosa Hospital of Viterbo in June 2022 with asthenia and abdominal pain. At physical examination, spleen enlargement was detected, with anemia (Hb 10.5 g/dL) and thrombocytopenia (48 × 10^9^/L). Abdominal echography confirmed spleen enlargement (20 cm of longitudinal diameter). Blood tests showed free light chain alteration with a *λ*/*κ* ratio of 800. Marrow aspiration showed 60% of λ‐restricted immature plasma cells: p53 expression was present in 91% of elements. Positron emission tomography/computed tomography (PET/CT) scan revealed multiple focal areas of increased metabolic activity in the bones and a widespread positivity of the spleen with focal areas of higher uptake. A diagnosis of MM with splenic EMD was made, and the Dara‐VMP regimen (daratumumab, bortezomib, melphalan, and prednisone) was started. After the first cycle of therapy, a marked reduction in spleen size was observed with an increase in both Hb level and platelet count. After the second cycle of therapy, however, there was evolution into plasma cell leukemia: the Vd‐PACE regimen (bortezomib, dexamethasone, cisplatin, doxorubicin, cyclophosphamide, and etoposide) was thus started, but after the second cycle, she died in October 2022 from septic shock and multiorgan failure.

**Conclusions:**

Our very rare case of ND/MM with spleen involvement confirms the aggressive behaviour of EMD, with negative prognostic factors (p53 mutation) and failure to frontline highly effective therapy. In the other few cases of spleen involvement reported, however, there were only scarce details about response: as a consequence, collection of similar cases is warranted to fully understand clinical features and possible alternative approaches for these extremely rare patients.

Multiple myeloma (MM) is a relatively common hematologic malignancy characterized by clonal plasma cell proliferation [1]. Generally, neoplastic plasma cells are restricted to the bone marrow: however, a sizeable rate of MM patients has extramedullary disease (EMD), defined as the presence of neoplastic plasma cell proliferation at distant sites from the bone marrow through hematogenous dissemination. This type of presentation accounts for about 1.7%–4.5% of the newly diagnosed MM and in 3.4%–10% of patients with relapsed or refractory disease (RR/MM) [2, 3].

When an EMD is present, it generally correlates with aggressive behavior: usually, an elevated LDH level is found in association with other negative prognostic factors, as del 17p or clonal mutations in some genes (TP53, RB1, FAK, and RAS) [4].

Herein, a very rare case of spleen involvement in a patient with newly diagnosed MM is reported.

## 1 Case Report

A 72‐year‐old female was admitted to the emergency department of Santa Rosa Hospital in Viterbo in June 2022, referring to asthenia and abdominal pain. Dyslipidemia, hypothyroidism in substitutive treatment, hysterectomy due to uterine fibromatosis, cardiac surgery for an aortic valve replacement, and a recent SARS‐CoV‐2 infection were reported in her medical history.

**FIGURE 1:**
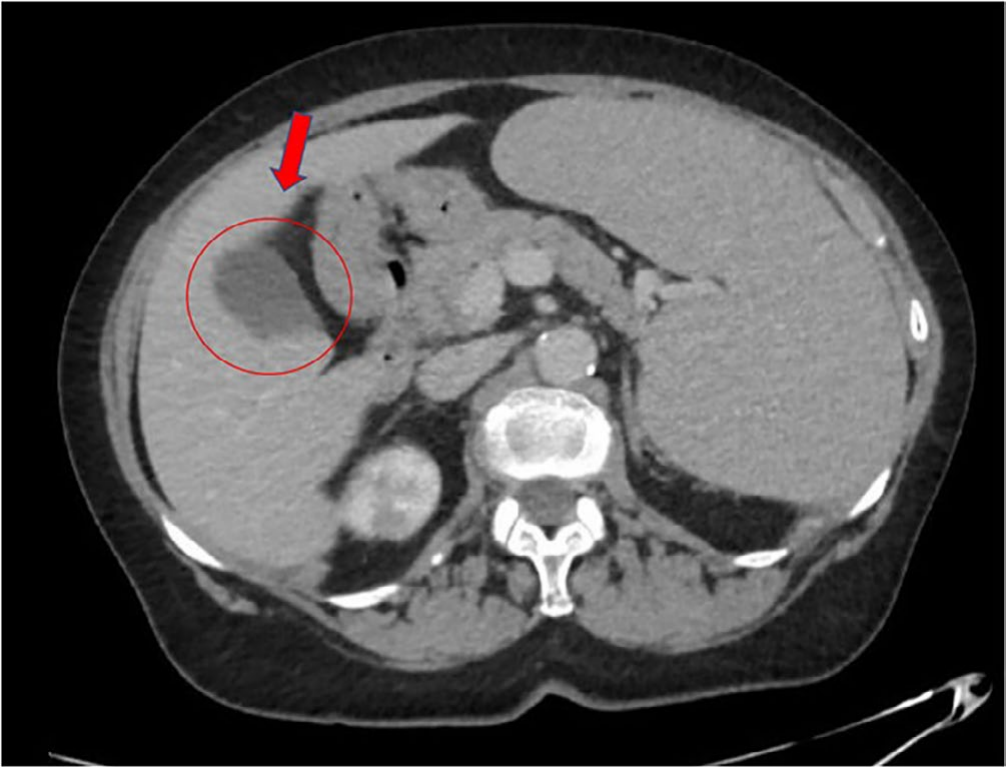
Abdominal tomography at diagnosis, showing splenomegaly with lower pole infarction (indicated by the arrow in the circle).

At physical examination, abdominal distension and spleen enlargement were detected: the splenic lower pole was palpable at 7 cm below the costal margin. Blood count showed anemia (Hb 10.5 g/dL) and thrombocytopenia (48 × 10^9^/L). Abdominal echography and computed tomography (CT) scan confirmed spleen enlargement (20 cm of longitudinal diameter) with an ischemic area at the lower pole and revealed small ascitic effusion (Figure 1).

**FIGURE 2:**
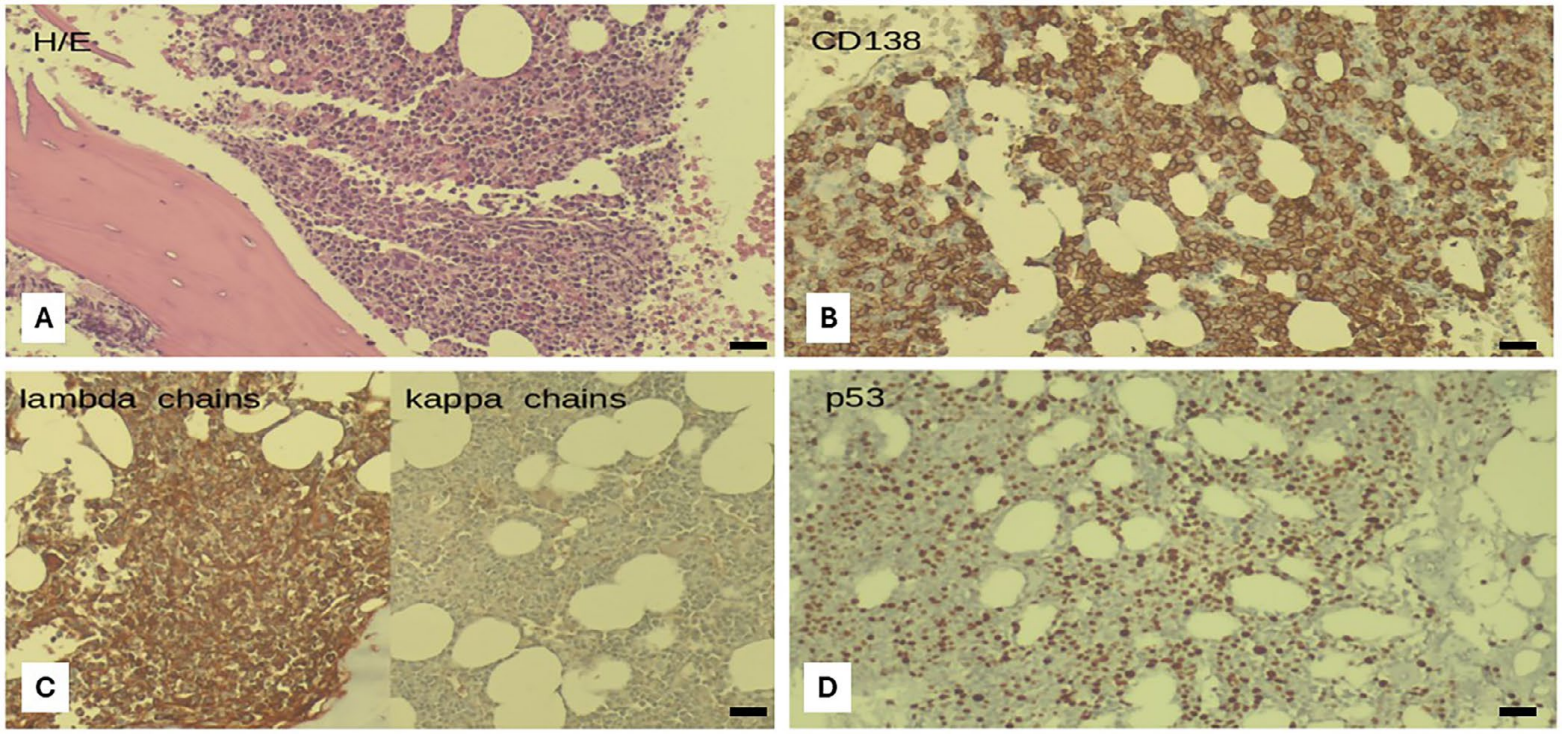
Bone marrow histology showing the following features: (A) hyper‐cellularity; (B) 60% of infiltration by plasma cells CD138+ and (C) Lambda+; (D) positivity of p53 (magnification ×10).

The blood tests showed elevated LDH, immunoglobulin level reduction (IgG 348 mg/dL, IgA 16 mg/dL, IgM 8 mg/dL), and a serological free light chain alteration (*κ* 3.6 mg/L—*λ* 2880 mg/L, with a *λ*/*κ* ratio 800). The bone marrow aspiration and biopsy showed heavy infiltration (60% of the total marrow cellularity) by λ‐restricted immature plasma cells (CD138+, CD20−, CD79a−, CD56−, cyclin D1−, CD3− at immunohistochemistry staining) (Figure 2).

**FIGURE 3:**
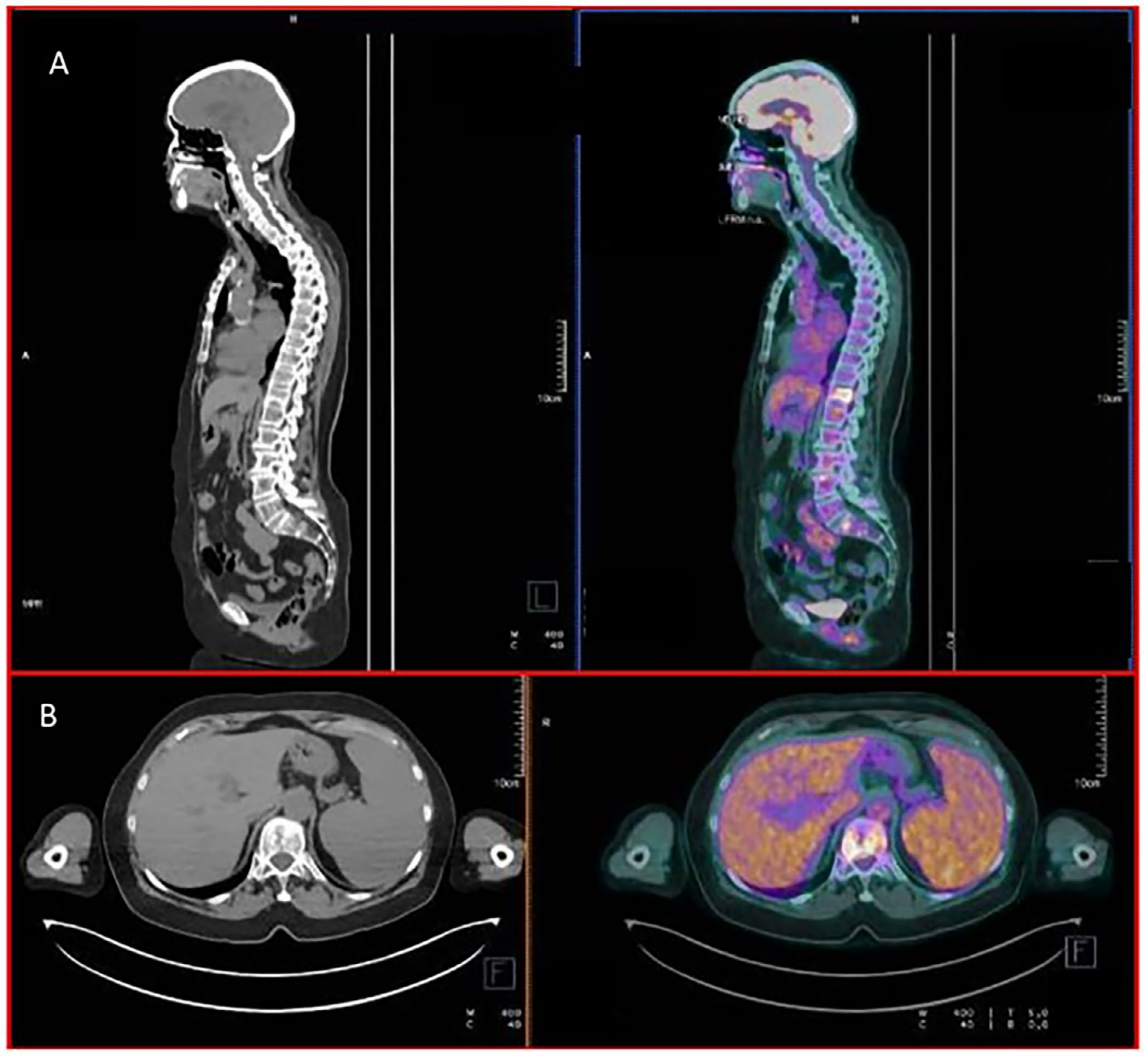
PET‐CT scan at diagnosis showing the following features: (A) Diffuse areolas of increased metabolic activity in some vertebral body; (B) Uptake of the splenic parenchyma with focal areas.

Fluorescence in situ hybridization (FISH) examination of the bone marrow plasma cells evidenced monosomy of chromosome 13, while high‐risk chromosomal abnormalities, including t(4;14), t(14;16), and 1q abnormalities, were absent; the p53 expression was present in 91% of elements. Magnetic resonance of the skeleton showed diffuse areolas of altered hypointense signal in T1 and T2, extending to the entire spine, pelvis, and proximal portions of the femurs; the larger ones were located in the vertebral bodies of D8 and D12, which were completely replaced, and at the proximal third of femoral shafts (longitudinal diameter up to 4 cm) (Figure 3A). To complete the staging, a total body positron emission tomography/CT (PET CT) scan was performed and confirmed the multiple focal areas of increased metabolic activity at the thoracic vertebrae D2, D8, D9, D12, L3, L4, sacrum, iliac bones bilaterally, left ilio‐pubic ramus, ischiopubic ramus, femur, and some right costal margins. In addition, there was a widespread positivity of the splenic parenchyma with the presence of many focal areas of higher uptake (Figure 3B).

**FIGURE 4:**
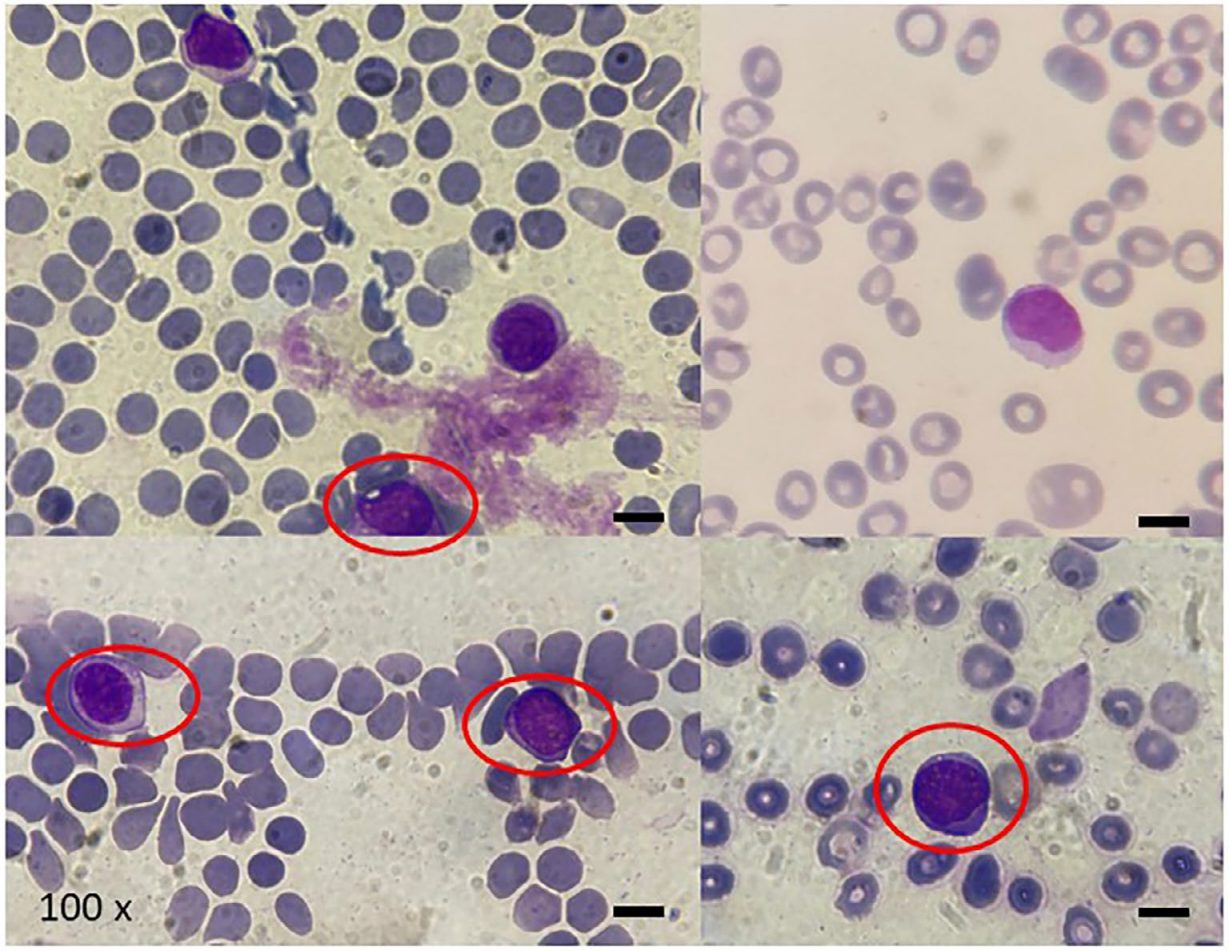
Peripheral blood smear at disease progression showing circulating plasma‐cells (indicated in the circles) (magnification ×100).

Diagnosis of EMD was thus performed, and the Dara‐VMP regimen (daratumumab, bortezomib, melphalan, and prednisone) was started. After the first cycle of therapy, a marked reduction in abdominal pain and in the spleen size (12 cm of longitudinal diameter) was observed, along with an increase in both the Hb level (11.0 g/dL) and in the platelet count (100 × 10^9^/L). After the second cycle of therapy, however, the EMD evolved into plasma cell leukemia (Figure 4).

**FIGURE 5:**
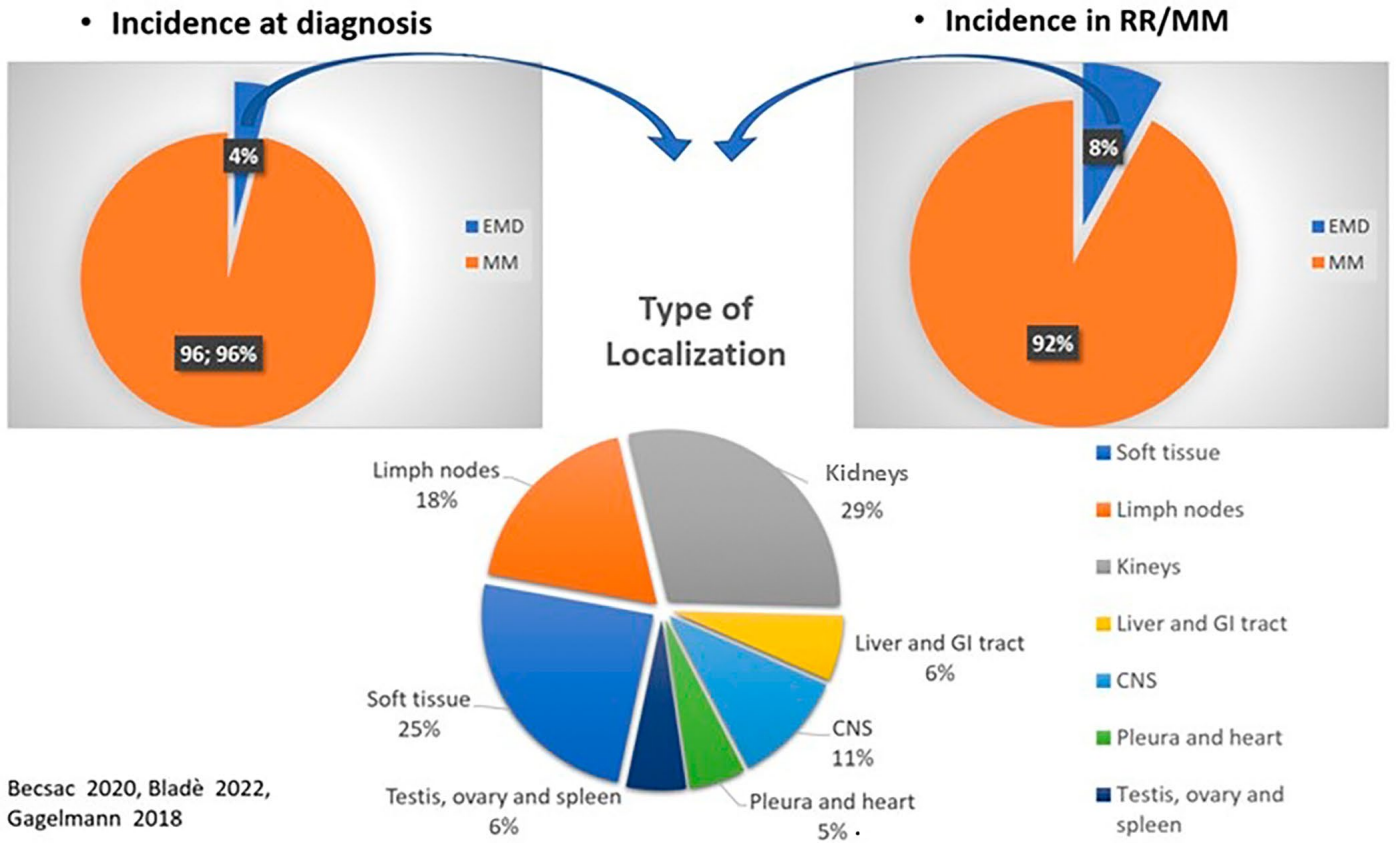
Incidence and type of tissue localization of extra‐medullary ND/MM and RR/MM.

The patient was hospitalized in October 2022 with pancytopenia (WBC 0.75 × 10^9^/L and 25% circulating peripheral plasma cells, Hb 7.6 g/dL, PLT 36 × 10^9^/L) and very high LDH value (2523 U/L): a new FISH and immunophenotypic analysis showed the same results as the initial diagnosis. Chemotherapy according to the Vd‐PACE regimen (bortezomib, dexamethasone, cisplatin, doxorubicin, cyclophosphamide, and etoposide) was started [5]. The patient received 2 cycles of therapy. The first cycle was complicated by two episodes of sepsis and cellulitis: before the initiation of the second cycle, the peripheral blood smear was negative for circulating plasma cells, with marked reduction in LDH levels (436 U/L). On Day 7 of the second cycle, she presented with fever and procalcitonin elevation (28.73 ng/mL) and a *Klebsiella pneumoniae* carbapenemase‐producing (KPC) was isolated from blood cultures. Septic shock supervened, and despite intensive treatment with fluids, vasoactive drugs, and antibiotics, on Day 13 she died from multi‐organ failure.

## 2 Discussion

The definition of EMD is quite controversial. Previously, the clinical presentation of EMD was defined as (1) a tumor mass located closer to the bone, with extension to the soft tissues and the bone marrow (bone‐related plasmacytoma), or (2) a tumor mass or a diffuse plasma cell infiltration in distant sites of the body not related to the bone [2, 6].

Most recently, in 2022, Joan Bladé et al. proposed to consider mainly the biological behavior and the prognostic features of the disease: the term EMD was limited to “an aggressive form of multiple myeloma characterized by the presence of soft‐tissue plasmacytomas as a result from hematogenous spread”, thus excluding bone‐related plasmacytoma from EMD definition [3].

As described above, the reported rate of patients presenting with EMD varies between 1.7% and 4.5% of newly diagnosed MM cases [3]. These variable frequencies are due to the different sites of the myeloma localizations, the different definitions of EMD, and the different diagnostic techniques used; for example, with an adequate technique of imaging (whole body‐MRI or PET/CT), the incidence of EMD raises approximately to 7% [7].

In the relapsed/refractory setting, the prevalence of EMD has been reported to be as high as 10%–30%; however, its real prevalence could be higher, taking into account that autoptic studies have revealed extramedullary involvement in approximately 70% of suspected cases, with up to 40% infiltration in non–bone‐related distant sites [3].

In 2018, the European Society for Blood and Marrow Transplantation reported on a retrospective trial involving 3744 MM patients, of which 3.7% presented extra‐skeletal involvement. The extramedullary sites involved were, in decreasing order of prevalence, kidneys (27.3%), skin (23%), lymph nodes (17.3%), central nervous system (10.1%), lungs and respiratory tract (6.5%), liver and gastrointestinal tract (5.8%), pleura and heart (5%) and spleen, ovaries, and testicles (5.3%) (Figure 5) [8].

The molecular mechanisms that could explain plasma cell hematogenous spread are not well defined: a putative role could be played by the altered expression of some molecules that promote adhesion of plasma cells to the bone marrow matrix (such as CD56 and P‐selectin), the lower expression of CD44 Ag that is involved in cellular adhesion, migration, and proliferation, and the increased activity of the angiogenesis‐promoting factors VEGF, CD31, and MMP‐9 [9]. In EMD, histological examination frequently shows an immature/plasmablastic appearance of neoplastic plasma cells [10]. All the previous features contribute to the lower progression‐free survival and overall survival of the EMD compared to “classical” MM, even in the era of novel agents.

Splenic infiltration at diagnosis is an extremely rare event; in Table 1 are summarized the few cases reported with some details in the literature [9, 11, 12, 13, 14, 15, 16]. Some features can be highlighted notwithstanding the rarity of such localization. First of all, the age of subjects with spleen involvement seems generally lower than the standard age of MM patients, and female gender seems prevalent; in addition, spleen enlargement, both isolated or associated with marrow infiltration, may often be complicated by pathological spontaneous spleen rupture at the onset of disease [9, 11, 14]. Our patient was the oldest and presented with a spleen involvement revealed at the CT‐PET scan, which showed a widespread uptake of 18F‐ FDG with focal areas of greater accumulation. Some direct mechanisms have been described to explain splenic enlargement in MM, such as extra‐medullary haematopoiesis, which is an uncommon finding in this disease, and the tumoral growth inside the splenic tissue. In most cases, the diagnosis was based on histological findings after splenectomy: on the contrary, in our case, the histological evaluation by fine needle aspiration was considered dangerous due to the incidental risk of spleen rupture and the high risk of bleeding owing to low platelet count.

**TABLE 1:** Main features of cases with multiple myeloma and spleen involvement reported in literature.

Authors (year)	Age	Sex	Diagnosis	Treatment	Response	Survival
Perry‐Thornton [11] (1989)	65	M	ND/SM IgG(K)	Splenectomy^a^	PR	> 2 years
Horny [12] (1992)	NR	F F	ND/SM IgG‐κ ND/SM IgG‐ƛ	Splenectomy	PD	NR
Colovic [13] (1998)	54	M	ND/SM IgG‐ƛ	Splenectomy ➔ VBCMP	CR	NR
Ho [14] (2008)	52	F	ND/MM	Splenectomy^a^➔ NR	NR	NR
Ryzhko [15] (2013)	52	F	ND/MM IgG‐κ	VD ➔ Splenectomy	PR	NR
Wang [16] (2015)	23	F	ND/SM	Splenectomy	CR	> 2 yrs
Abreu [9] (2019)	50	F	ND/MM IgG‐κ	Splenectomy^a^ + VD‐PACE ➔ CTD‐ABMT	PR	NR
Our case	72	F	ND/MM IgG‐κ	DaraVMP ➔ VD‐PACE	PD	6 months

^a^ Spontaneous rupture of spleen.

Abbreviations: ABMT = autologous bone marrow transplantation; BCNU, cyclophosphamide, melphalan, prednisone; CR = complete response; CTD = cyclophosphamide, thalidomide, dexamethasone; Dara‐VMP = daratumumab, bortezomib, melphalan, prednisone; ND/MM = new diagnosis/multiple myeloma; ND/SM = new diagnosis/solitary myeloma; NR = non‐reported; PD = progressive disease; PR = partial remission; VBCMP = intermittent vincristine; VD = bortezomib, dexamethasone; VD‐PACE = bortezomib, examethasone, cisplatin, doxorubicin, cyclophosphamide, etoposide.

The following important lessons can be drawn from our case: first of all, it confirms the aggressive behavior of EMD, sharing negative prognostic factors, such as p53 mutation, and failing to respond to frontline therapy, even when new highly effective drug associations were employed. In addition, our report highlights that spleen involvement in patients with MM is rare but can occur and should be considered as a possible site of disease, especially in high‐risk subjects: due to the rarity of cases with spleen involvement reported in the literature, however, there are at present only scarce details about response to treatments and survival. As a consequence, the collection of other cases is warranted to fully understand main clinical features and possible alternative therapeutic approaches for these patients who continue to be an unmet clinical need.

## Author Contributions

**Alessia Fiorini:** conceptualization (lead), data curation (equal), validation (equal), writing – original draft (equal). **Maria Gabriela Chavez:** conceptualization (supporting), data curation (equal). **Valentina Panichi:** data curation (equal), validation (equal). **Marco Dell'Aquila:** data curation (equal), resources (equal), writing – original draft (equal). **Valentina Ranucci:** data curation (equal), validation (equal). **Daniele Remotti:** data curation (equal), supervision (equal). **Michela Tarnani:** data curation (equal), validation (equal). **Marco Montanaro:** conceptualization (equal), formal analysis (equal), validation (equal), writing – original draft (equal). **Roberto Latagliata:** conceptualization (supporting), formal analysis (equal), supervision (lead), writing – original draft (equal). **Alessandro Andriani:** conceptualization (equal), supervision (equal), validation (equal), writing – original draft (equal).

## Consent

Written informed consent for the publication of case details and use of images was obtained from the patient's daughter.

## Conflicts of Interest

The authors declare no conflicts of interest.

## Data Availability

The data that support the findings of this study are available from the corresponding author upon reasonable request.
